# Youth underrepresentation as a barrier to sexual and reproductive healthcare access in Kasulu district, Tanzania: A qualitative thematic analysis

**DOI:** 10.1007/s00038-020-01367-6

**Published:** 2020-04-09

**Authors:** Respicius Shumbusho Damian, Henry Zakumumpa, Sharon Fonn

**Affiliations:** 1 Department of Political Science and Public Administration, College of Social Sciences, University of Dar es Salaam, Dar es Salaam, Tanzania; 2 School of Public Health, Makerere University, Kampala, Uganda; 3 School of Public Health, University of the Witwatersrand, Johannesburg, South Africa

**Keywords:** Youth representation, Sexual and reproductive health, Health facility committees, Healthcare access, Tanzania

## Abstract

**Objectives:**

Representation of the key groups in community-level healthcare decisions is a prerequisite for accountable and responsive primary healthcare systems. However, meaningful representation requires both the presence of individuals who represent the key community groups and their capacity to influence the key healthcare plans and decisions. Our study explored how the underrepresentation of the youth in health facility committees, the decentralized community- and facility-level healthcare decision-making forums affects youth access to sexual and reproductive health services.

**Methods:**

A multisite case study involving focus group discussions, interviews, and meeting observation was conducted in eight primary healthcare facilities in Kasulu, a rural district in Tanzania. Inductive thematic analysis was used to identify the key emerging themes.

**Results:**

Five major themes were identified in connection with youth underrepresentation and limited access to sexual reproductive health as a ‘taboo’ phenomenon in the communities. These were: numbers do not matter, passive representation, sociopolitical gerontocracy, economic vulnerability, and mistrust and suspicion.

**Conclusions:**

Gradual emancipatory and transformative efforts are needed to normalize the representation of the youth and their concerns in formal community-level decision-making institutions.

## Introduction

The population of Africa is growing youthful. In 2015, the United Nations estimated that 75% of the African population is below 35 years and nearly 20% of the population is aged between 15 and 24. Whereas the world’s youth population is expected to decrease from 16 to 14% by 2050, in Africa it is expected double (United Nations 2014). One of the reasons behind this growth is the higher fertility rate, which is 5.02 births per woman, declining maternal mortality rates (MMR), and gains in child survival. These projections present a message that youth access to health care will be an important aspect of the pursuit of Universal Health Coverage (Manyeh et al. 2018). In other words, the pressure on health systems to meet the important, but currently unmet youth healthcare needs will be unescapable (Finlay et al. 2020). Sexual and reproductive health (SRH) is one of (if not) the most important healthcare needs of the youth.

Being a critical mass, effective representation is and the only feasible tool that would allow the youth to influence healthcare decisions for improving health services in low-income communities (Rifkin 2014). However, effective representation is a two-dimensional concept. It has a numerical dimension and an issues’ dimension. The numerical dimension, which is the first, entails the physical presence of the youth in planning and priority setting forums such as Health Facility Governance Committees (HFGCs) (Plotke 1997). The issues’ dimension as the second relates to the extent to which such forums are receptive and willing to attend to youth health concerns (Ensor and Cooper 2004). It also relates to the perceived influence of the represented and their representatives on the actions and decisions of healthcare decision-making and implementing authorities (Rubino-Hallman 1998). Therefore, effective representation empowers the communities to influence healthcare plans and decisions in favour of the youth (Sleath et al. 2018) and allows the youth to voice for inclusive and responsive healthcare plans and decisions that improve availability and accessibility of priority and high-demand services such as SRH services (Coyne and Gallagher 2011). On the other hand, underrepresentation which entails both the absence of the youth on decision-making forums and constrained freedom of the youth to decide how the existing healthcare providers should serve them affects both the availability and accessibility of important healthcare services including SRH services (Liebenberg 1999).

Tanzania like many other low- and middle-income countries (LMICs) has put in place institutional arrangements for ensuring that the voices of all groups in communities are represented in primary healthcare (PHC) decision-making. Health Facility Governance Committees (HFGC) were introduced in all PHC facilities since 1999 as the community and facility-level accountability forums for improving the representation of community groups in decision-making (Borghi et al. 2013; Macha et al. 2011). Following the adoption of a community-financed PHC system in 2001, HFGCs have served as key platforms through which groups in communities can amplify their voices to demand availability and accessibility of health services (Sarfati et al. 2018). The representation of groups such as women and youth is vital for making their health concerns heard and attended by policy-makers and service providers.

In Tanzania, SRH is a component of an essential healthcare package. All PHC facilities are required to make SRH a part of their priorities, and it is a mandate of HFGCs to ensure the availability of SRH services in their facilities. There are some improvements in service availability and accessibility that appear to correspond with the recent efforts to ensure effective representation of different community groups in PHC governance. However, these improvements are not reflected in terms of youth access to SRH services. With the assumption that the lack of youth control over healthcare plans and decisions contributes significantly to the limited availability and accessibility of youth-targeted health care, we sought to explore how underrepresentation in decision-making through HFGCs affects youth access to SRH services in the context a rural remote community.

## Methods

### Study design

We analysed qualitative data from a broader mixed-method study, a Ph.D. project by the first author. The youth were defined as persons between 15 and 35 years old based on the National Youth Development Policy. The case-study design was employed to get in-depth insights (Stake and Savolainen 1995; Yin 2017) and experiences of the youth and other key stakeholders who were participating in HFGCs. We were also interested in how social norms and beliefs affected collective voicing by the youth.

### Study setting and sample

Kasulu district has the highest rurality index (98.2%) in Tanzania. Eight PHC facilities in 8 villages from 4 subdivisions were selected from a total of 32 PHC in 66 villages. Systematic random sampling was used to select PHC facilities. The facility was included if it had an operating facility committee. A total of 137 respondents participated in the study including 106 (32 youth) and 74 HFGC members who participated in focus group discussions (FGDs) and 31 PHC stakeholders from facilities and village governments who were interviewed. Table 1 summarizes the respondents by category.

**Table 1:** Selected respondents by primary healthcare facilities, categories, and gender (Tazania-2017)

	Committee FGDs			Youth FGDs			Respondent for Key Informant Interviews					Total
	M	F	Total	M	F	Total	FMs	FHWs	VCPs	VEOs	Total	
Bugaga dispensary	6	4	10	2	2	4	1	1	1	1	4	18
Kaguruka dispensary	7	3	10	2	2	4	1	1	1	1	4	18
Lalambe dispensary	4	5	9	2	2	4	1	1	1	1	4	17
Mugombe dispensary	3	4	7	2	2	4	1	1	1	0	3	14
Nkundutsi dispensary	5	4	9	2	2	4	1	1	1	1	4	17
Nyakitonto Health Centre	9	4	13	2	2	4	1	1	1	1	4	21
Rungwe Mya dispensary	5	3	8	2	2	4	1	1	1	1	4	16
Titye dispensary	5	3	8	2	2	4	1	1	1	1	4	16
Total	44	30	74	16	16	32	8	8	8	7	31	137

*HFGC* Health Facility Governance Committee, *M* male, *F* female, *FM* facility manager, *FHW* female health worker, *FGD* focus group discussion, *VCP* village chairperson, *VEO* village executive officer

## Data collection

We combined FGDs, semi-structured interviews, and meeting observations between August 2015 and July 2017. We interviewed one facility manager, female health worker, and village officials for each facility on how HFGCs represented youth SRH concerns. Youth FGDs elicited experiences of male and female youths regarding their influence on the committee decisions and its effect on SRH services availability and accessibility. Discussions with HFGCs focused on how the committees were dealing with youth SRH service concerns. We also attended and observed7 HFGC meetings. The first author moderated all the FGDs by introducing the discussions, asking probing questions, and encouraging participation. A trained female research assistant moderated the female youth FGD and participated in all the committee FGDs. Each of the discussions lasted between 60 and 90 min. Apart from getting shared understanding, FGDs were used to validate the data from interviews and meeting observations (Denzin and Lincoln 2011; Morgan 1996).

Each interview took an average of 50 min. We used an interview guide with 11 open-ended questions that focused on: how the formulation process ensured inclusion of youth, representation of youth concerns through the committees’ activities, and the general perceptions of youth representation and accessibility of SRH services. All the interviews were conducted by the first author who met the interviewees in their offices. The questions were translated into Kiswahili, which is understood by both the interviewer and interviewees and audio-recorded after getting verbal consent. The audio files from FGDs and interviews were saved on a secured computer and backed up on an external hard disk before the analysis.

### Data analysis

We were guided by a constructivist paradigm, which attempts to understand meanings in the existing context (Joniak 2005). Interviews and FGDs were transcribed verbatim, translated back into English, and coded inductively using NVivo version 10 software. We used a stage-by-stage procedure to identify emerging themes (Ryan and Bernard 2003), which were classified and combined to get more relevant, coherent, and inclusive themes. Attention was paid to the most recurring ideas and expressions as constituting our major themes.

## Results

Five major themes were identified in connection with youth underrepresentation in the context of HFGCs and how it affected youth access to SRH services in the study communities. These were: numbers do not matter, passive representation, sociopolitical gerontocracy, economic vulnerability, and mistrust and suspicion. We elaborate on them in the next five subsections.

### Numbers do not matter

Considering all the two dimensions of representation, namely numerical and issue representation, it was revealed that HFGCs were not providing effective representation of the youth. In addition to a substantively smaller number of representatives, active participation of the youth in HFGC decisions was rare. Therefore, there was underrepresentation than what the numerical representation dimension revealed. We noted that looking at the numbers of the youth on the committees could lead to a fallacious conclusion about the actual representation status since many of the representatives in the young age group were appointed to represent the interests of different social-economic groups in the communities. For instance, 24 percent of the representatives were under 35. However, more than half of them were primarily elected to the committees to represent the interests of private providers, women, and saving and credit associations.

‘It came accidentally that I am young, but here I am standing on behalf of the drug shop operators. Even if I get older than 35, I will be here’ [Female Youth Group).

‘We are young by age, but the groups we represent are diverse in terms of age. The SACCOs have women, young people, and all those who can save and borrow’. [Male Youth Group).

Therefore, we logically noted that representation through numbers constituted a representation fallacy.

### Passive representation

We further noted from interviews and FGDs that the presence of young people on the committees was not a guarantee that youth contributed to healthcare plans and decisions. Youth were labelled as passive participants who brought no issues on the agenda. They were claimed to be silent on youth concerns including SRH services of which the decisions were made by socially matured members. Interviews with facility managers and village officials revealed that communities considered HFGC meetings not to be the right places to discuss SRH issues, which was an attempt to avoid the ‘sex taboo’ which restrains sex-related talks by young people, especially in front of the elders.

‘When we sit down to identify discuss, some of us are very young. Some are our daughters and sons. How can we talk about sex things?’ (Interview with Village Chairperson)

‘We know some of the young people use them [SRH services], but we cannot entertain them because the committee members respect each other. Some of the members share the age with our fathers and mothers’ [HFGC FGD].

In connection with the passive representation, the notions that communities attached to the ‘sex taboo’ affect not only the voices of the youth in the committees but also the willingness of service providers to provide SRH education and related services such as condoms and female contraceptives in open public places. This was revealed by different respondents,

‘The condoms are there, but nobody will come and ask for them. They think the dispensary is there for treating patients and not distributing the condoms’ [Interview, Village Chairperson]

‘When you go there and take condoms. The nurse will look at you and tell the whole village. I can take them, but I don’t want the nurse to know it’ [Male youth FGD]

‘I know when I want a pregnancy test I could get it in the dispensary, but how do I start? How do I tell the nurse I am pregnant and I need an abortion? The story will go all the way around the village’ [Female youth FGD]

Neither the youth nor their representatives on the committees can actively voice out to demand such services. This makes the representatives of the youth passive and less capable of having a substantive impact on decisions and practical actions that aim at making SRH services available and accessible. Generally, the commonly shared conception of youth and SRH services constrain the representation of youth voices and influence on health services access.

### Sociopolitical gerontocracy

Social gerontocracy, the rule by the old men, came out as an important theme that connects youth underrepresentation and access to SRH services. Age and gender determined the right to influence decisions at all levels. In all the study communities, age and gender notions shaped the perceptions of who is entitled to make decisions for the rest of the communities. Decision structures are hierarchically constructed around age and gender with all men at the top and all women at the bottom. We noted regular use of the word ‘*wakubwa*’ (which is a Kiswahili word for a grown-up, matured, or big person) in all the FGDs referring to those who make key decisions. This word is mostly associated with seniority. In addition to seniority, gender was found to play a decisive role. The male elders are entrusted with decision-making powers and are considered the only source of wise and right decisions.

We observed that men’s decision-making power was a natural entitlement and is constructed through day-to-day life practices. For example, women leave chairs to let men sit. Food is usually served to men first. In all the FGD meetings, women appeared to be passive recipients of decisions even on matters that directly affected them such as maternal health care. These practices start at a family level, but widely dominate decision-making in governance platforms including HFGCs and thus placing the youth, especially young girls as powerless and unable to choose and demand SRH services in their communities.

‘When you are there [in the meeting] as a young person, your role is to keep quiet and listen to what they [old men] say. If you pretend to know you will be in trouble' [Male youth FGD].

‘We are not there to challenge the wisdom of the elders. If we start thinking we know everything we will soon be in trouble. What is not good for them [old men] cannot be good for us’ [HFGC FGD]

In addition to age and gender, sociopolitical gerontocracy is enhanced by other factors such as wealth, fame, marital status, and attachment to loyal families. Young persons who enjoyed these socioeconomic statuses appeared to have a privilege to speak and influence decisions. For instance, it is unusual to listen or pay attention to the advice of a man who is not married regardless of his age.

The sustenance of gerontocracy in the communities rests on the point that both the youth and the elders have accepted the status quo. They largely agree that the elders have a positive aim, which is protecting the positive norms of the communities. This affects the influence of the youth on SRH service plans and decisions on one hand and publicly availability and accessibility of such services in the study communities.

### Economic vulnerability

We found from both interviews and FGDs that economic vulnerability among the youth undermined the influence on decisions and access to SRH services. Economic power suggestively determined whether someone would be elected to HFGCs. The youth who owned property such as big land, modern houses, and businesses or had financial success were preferred when formulating the committees. However, very few of the youth in the study communities had such endowments. Related to this, election to HFGC is largely considered to be a blessing that makes a young person likely to enjoy economic and political privileges that the communities have including land allocation by a village authority or a parent as well as appointment by a political party to contest for a political office. We learned that the desire to preserve these potentials made the youth who were on the committees incapacitated in terms of standing for the health needs of the young people including SRH services.

‘Many of us expect to get land from either our parents or the village government. If you talk too much it becomes difficult' [Male youth FGD].

‘The whole village knows who is humble and who is disrespectful. So, if you act stubbornly in the name of claiming the rights to health, you will die of hunger. When you look for a farm job everybody will say no’ [HFGC FGD].

In other words, economic vulnerability is connected with virtues of being passive, voiceless, and sarcastically humble behind which there is a development of a civic culture of parochialism and patronage. These virtues were generally found to make the youth substantially underrepresented and incapable of influencing authorities to make SRH services sufficiently available and thus accessible.

### Mistrust and suspicion

The other important theme, which was identified as a barrier to representation and access to SRH services, was the widespread mistrust and suspicion. We found that the youth the trust of the youth all those who are connected to government authority including village officials and health workers are very limited. The notion *wakubwa’* as it was introduced in connection with gerontocracy similarly symbolizes authority. There was a perception in all study communities that representative institutions such as HFGCs represent the personal agenda of individuals, which are mostly political rather than addressing youth health concerns including SRH services.

‘What you are saying is the opposite. They [those in authority] talk and agree that sexual education should not be provided to the youth. They should not fool us. We areS not small children who do not know. They are there for their businesses and not for us’ [Male youth FGD]

‘…We don’t think they [HFGCs] will succeed because they are being misused. Instead of working on problems of this hospital (dispensary) they are used by few people for their own interest’ [Female youth FGD]

The dominant mistrust and suspicion were found to undermine the whole idea of representation and constrain access to services because the voices of the youth are systemically suppressed both within and outside the HFGCs.

As a manifestation of mistrust and suspicion, we found the youth to have developed some coping mechanisms that helped them to share their concerns while avoiding authorities. The most common was the use of symbolic Kiswahili language terms that were used to refer to condemned actions and inactions of the authorities. A good example of these words is ‘*tumeliwa*’ (translated as we have been eaten*)*. The hidden meaning that is shared through this word that they have already been manipulated by the authorities. Another example is ‘*tuwatose’* (translated as let us sink them). This is used to share a proposal that they should not cooperate with the authority in a matter in question). The third and last example is ‘*watu wao*’ (translated as their people*).* The youth use this phrase to refer to people whom they suspect to be colluding with authorities. The phrase is commonly used by the youth to refer to their fellow youth who hold representative positions but thought to serve the interests of the authorities rather than the youth.

Mistrust and suspicion affect both representation and youth access to the services. On the first hand, the whole idea of representation is undermined because of the division that exists between the youth, their representatives, and the authorities. On the second hand, pressures to make the authorities and communities realize the pressing need of SRH services and thus relax the social-cultural norms to allow for increased availability and accessibility of the services in the communities are passively suppressed.

## Discussion

Revealed in this respect is that having sufficient numbers of young people in decision-making may not necessarily mean representation of the youth. Unlike other community groups, which have special windows for representation on the committees, the youth access the committees as representatives of other community groups in their communities. This increases the chances of underrepresentation right from the formulation of HFGCs. The findings also reveal that the numerical dimension of representation bears a fallacy in it since the committees do not have internal mechanisms to make sure that the voices of the youth are tapped into the discussions and decisions of the committees. The findings, like previous studies (Tylee et al. 2007; Sleath et al. 2018), reinforce the need to focus on the extent and existing mechanisms that allow the youth to influence the decisions and actions related to making SRH services acceptable in the communities and thus available and accessible (Skinner et al. 2003).

Our study was an attempt to explore how the underrepresentation of the youth in decision-making through HFGCs affects youth access to SRH services in rural PHC in Tanzania. We contributed to the conceptual understanding of representation by revealing that a mere presence of representative participatory organs and having individuals participating in the activities of these organs should be taken as a means rather than an end within itself. The findings challenge the assumption that the existence of decentralized community- and facility-level decision-making arrangements such as HFGCs would automatically improve youth representation and subsequently the accessibility of SRH services (Frumence et al. 2014; Kilewo and Frumence (2015); URT 2001). It is worth learning that each community has a system of cultural norms through which policies are assigned meanings. The attempt by communities to preserve their norms and traditions may obstruct the implementation of legally approved healthcare interventions that seek to address critical health problems of the communities.

As some of the previous studies have already revealed (Campero et al. 2011; Frumence et al. 2014; Damian 2018), effective representation of community voices and health concerns needs to entail at least three major dimensions. The first dimension is the physical presence of representatives of interested community groups in decision-making organs. The second dimension is the process whereby the interests and concerns are articulated and accommodated as part of the collective concerns of the communities. The third is the sociopolitical environments that sanction representation of the most pressing concerns and the concerns themselves as socially acceptable and legitimate. Therefore, the effect of youth representation on the access to the SRH services would depend on how many youths are elected to the committees to represent the youth themselves, the extent to which the representatives can influence the decisions of the committees, and the social interpretation and receptivity of the raised health concerns not only by the decision-makers but also the communities.

Sociopolitical gerontocracy and related socioeconomic relations such as patronage and patriarchy contribute to vulnerability and deprivation of opportunities for the youth and women (Campero et al. 2011; Gilmour et al. 2000). These systems continue to shape the relations in many communities despite the struggle to match with the international agenda that gives priority to the empowerment of all social groups, equitable access to services, and accountable decision-making health services delivery. The coexistence between the ongoing global health promotion initiatives and these sociopolitical values makes the realization of universal coverage a well desired but challenging endeavour (Damian 2018). Related to SRH, the coexistence of these beliefs and the formal SRH care interventions limits the pace of implementing youth-friendly SRH programs in community settings (Tylee et al. 2007). This means that the measures to meet the unmet demand for youth SRH services need to be culturally sensitive. The reason is that the communities see the importance of both preservation of the social-cultural norms and adopting the modern modes of governance that entails empowering the traditionally powerless populations such as the youth and women to participate in decision-making and improved access to health services.

In connection with previous studies (Dunne et al. 2017; Henderson et al. 2017), our findings insist on three important messages regarding youth representation in PHC decisions and access to SRH services. First, effective representation is more than physical access to decision-making institutions. It entails developing capabilities of both the represented groups and their representatives to challenge the social norms and beliefs that constrain their individual and collective voices. Second, the youth as a group need special attention since their identity and rights can be confined to other social, economic, and political groupings, which hardly represent their concerns. With this, even numerical representation would always remain superficial if there is no well-defined quota for the youth.

Taking this view, the designers of community-level participatory healthcare priority-setting, planning, and decision-making institutions such as HFGCs need to consider the possible constraining forces of the dominant existing social and cultural belief systems. These systems may pose potential restrictions despite the formal mechanisms for representation of the youth in key healthcare decision-making institutions. Accordingly, learning from previous studies on youth empowerment (Herbst et al. 2017; Skinner et al. 2003), an approach that integrates promoting collective voices to allow the youth to influence the key primary healthcare decisions, and understanding and eliminating the social and cultural barriers that limit effective representation and unconstrained access to SRH services is required. Sensitizing the communities to understand the pressing need of youth SRH services and the best ways of providing such services without undermining the positive cultural norms of the communities is a potential challenge of community-level and primary healthcare systems’ designers.

Despite the depth of our analysis, two limitations need to be acknowledged. First, both interviews and FGDs were based on the participants' experiences and perceptions. With this, the correctness, validity, and reliability of all the responses could not be guaranteed. Second, given the limited geographical coverage of our study area, the generalizability of our study findings may be limited to the context of the study communities. However, the lessons from this study may useful for understanding how social-cultural norms shape representation in community-level decision-making institutions and the subsequent effect of the conflict between social-cultural and healthcare promotion interventions on service delivery and access to sensitive healthcare services such as SRH.

### Conclusion

Youth access to sexual and reproductive health services appears to be an important, but challenging task. The ongoing efforts to empower different social-economic groups to participate in and influence healthcare decisions appear to be one of the most feasible ways of increasing access to group-specific healthcare needs as it is in the case of youth sexual and reproductive health care. Unlike other community groups such as women, both representation and access to sensitive healthcare services such as SRH are challenged by the fact that the existing community-level representative decision-making arrangements do not facilitate the effective representation of youth SRH concerns because of the limited social acceptability of SRH services.

Our overall conclusion is that meaningful and effective representation of the youth in PHC decision-making that may have a positive implication on the accessibility of SRH services needs to go beyond having the young people elected on the committees. It needs the arrangements in place to ensure that the youth have a special window and are empowered to articulate pertinent health concerns and the concerns are legitimately channelled into healthcare plans and decisions. Overall, effective representation of the youth and improved access to SRH services requires an ongoing commitment to social emancipation interventions to increase community awareness of the inevitable need for youth SRH services in modern communities. This is a prerequisite for resolving the contradiction that exists between SRH services and the social-cultural notions regarding the position, right, and responsibility of a young person.
